# PEG35 and Glutathione Improve Mitochondrial Function and Reduce Oxidative Stress in Cold Fatty Liver Graft Preservation

**DOI:** 10.3390/antiox11010158

**Published:** 2022-01-14

**Authors:** Raquel G. Bardallo, Idoia Company-Marin, Emma Folch-Puy, Joan Roselló-Catafau, Arnau Panisello-Rosello, Teresa Carbonell

**Affiliations:** 1 Department of Cell Biology, Physiology and Immunology, Faculty of Biology, Universitat de Barcelona, 08028 Barcelona, Spain; rgomezbardallo@ub.edu (R.G.B.); i.company-marin@aston.ac.uk (I.C.-M.); 2 Experimental Pathology Department, Institut d’Investigacions Biomèdiques de Barcelona—Consejo Superior de Investigaciones Científicas (IIBB-CSIC), 08036 Barcelona, Spain; emma.folch@iibb.csic.es (E.F.-P.); jrcbam@iibb.csic.es (J.R.-C.); arnau.panisello@iibb.csic.es (A.P.-R.); 3 Institut d’Investigacions Biomèdiques August Pi i Sunyer (IDIBAPS), 08036 Barcelona, Spain

**Keywords:** antioxidants, glutathione, Nrf2, OXPHOS, polyethylene glycol, UCP2, IGL-2

## Abstract

The need to meet the demand for transplants entails the use of steatotic livers, more vulnerable to ischemia-reperfusion (IR) injury. Therefore, finding the optimal composition of static cold storage (SCS) preservation solutions is crucial. Given that ROS regulation is a therapeutic strategy for liver IR injury, we have added increasing concentrations of PEG35 and glutathione (GSH) to the preservation solutions (IGL-1 and IGL-2) and evaluated the possible protection against energy depletion and oxidative stress. Fatty livers from obese Zücker rats were isolated and randomly distributed in the control (Sham) preserved (24 h at 4 °C) in IGL-0 (without PEG35 and 3 mmol/L GSH), IGL-1 (1 g/L PEG35, and 3 mmol/L GSH), and IGL-2 (5 g/L PEG35 and 9 mmol/L GSH). Energy metabolites (ATP and succinate) and the expression of mitochondrial oxidative phosphorylation complexes (OXPHOS) were determined. Mitochondrial carrier uncoupling protein 2 (UCP2), PTEN-induced kinase 1 (PINK1), nuclear factor-erythroid 2 related factor 2 (Nrf2), heme oxygenase-1 (HO-1), and the inflammasome (NLRP3) expressions were analyzed. As biomarkers of oxidative stress, protein oxidation (AOPP) and carbonylation (DNP derivatives), and lipid peroxidation (malondialdehyde (MDA)–thiobarbituric acid (TBA) adducts) were measured. In addition, the reduced and oxidized glutathione (GSH and GSSG) and enzymatic (Cu–Zn superoxide dismutase (SOD), CAT, GSH S-T, GSH-Px, and GSH-R) antioxidant capacities were determined. Our results showed that the cold preservation of fatty liver graft depleted ATP, accumulated succinate and increased oxidative stress. In contrast, the preservation with IGL-2 solution maintained ATP production, decreased succinate levels and increased OXPHOS complexes I and II, UCP2, and PINK-1 expression, therefore maintaining mitochondrial integrity. IGL-2 also protected against oxidative stress by increasing Nrf2 and HO-1 expression and GSH levels. Therefore, the presence of PEG35 in storage solutions may be a valuable option as an antioxidant agent for organ preservation in clinical transplantation.

## 1. Introduction

The increasing incidences of obesity and unhealthy lifestyles in the world population are both responsible for the metabolic syndrome and the consequent accumulation of fat in the liver. Given the global shortage of livers for transplantation, studies on the conditions that allow the use of steatotic livers are needed to broaden donation criteria [1]. However, the use of steatotic liver grafts and therefore suboptimal increases the risk of later impaired function leading to cell necrosis, which is caused by the higher vulnerability to suffer an ischemia-reperfusion (IR) injury [2,3].

So far, the most widely used preservation method has been static cold storage (SCS). The purpose of the researchers is to find and define the optimal composition of commercial solutions used during cold ischemia, so that the functionality of the stored organ is maintained, ensuring the viability of the steatotic organ after reperfusion [4].

Among the most used solutions for liver transplantation is the Institut Georges Lopez-1 (IGL-1) solution [5,6]. IGL-1 contains polyethylene glycol (PEG) with a molecular weight of 35 kDa (PEG35), glutathione (GSH), and a higher concentration of Na^+^ in relation to that of K^+^. PEG is a variable-weight and neutral polymer with high solubility, due to its hydrophilicity and low toxicity, so it does not trigger an immunological response [7]. It protects against necrosis, prevents oedema formation and contributes to cell membrane stabilization and cytoskeleton integrity maintenance [8]. PEG helps to repair and maintain multiple cell structures, such as the plasma membrane in hypoxic conditions, as it happens in IR procedures [4]. Those facts entail several beneficial effects for IGL-1 compared to other solutions [9,10,11], both in healthy and steatotic livers. The benefits of the IGL-1 solution (1 g/L PEG35 and 3 mmol/L GSH) include the prevention of liver damage [12] and lipid peroxidation [13,14]. We recently used a new solution, IGL-2, (5 g/L PEG35 and 9 mmol/L GSH). The detailed compositions of IGL-1 and IGL-2 have been described in Bardallo et al. [15]. Our previous results showed that IGL-2 reduces fatty liver damage due to cold storage. Furthermore, we observed an increase in the mitochondrial expression of ALDH2, which could act as a guardian of the overproduction of reactive oxygen species (ROS), therefore protecting the steatotic liver graft from the damaging effects of cytotoxic aldehydes.

Since IR injury will significantly determine the functionality of the fatty liver graft after transplantation, it is necessary to explore the cellular mechanisms that lead to tissue damage. Among them, it is known that the mitochondrial response during ischemia stimulates ROS production and reduces adenosine triphosphate (ATP) levels, which have been identified as the main cause of reperfusion damage [16], by producing oxidation and tissue inflammation. Our objective is to address whether the beneficial mechanisms of the addition of PEG35 and GSH in storage solutions may be due to the maintenance of mitochondrial function and the modulation of the redox state in cold fatty liver preservation.

Energy metabolites, mitochondrial oxidative phosphorylation complexes, mitophagy, oxidative stress, and antioxidants were analyzed. We found that liver grafts preserved in IGL-2 maintained mitochondrial integrity and redox status; thus, this solution would be recommended for clinical liver transplantation purposes.

## 2. Materials and Methods

### 2.1. Animals and Liver Isolation and Perfusion

Male homozygous obese (Ob) Zücker rats from Charles River (France), aged 16–18 weeks, were used. Zücker rats constitute a well-characterized model of nutrition-induced obesity, closely simulating the most common cause of steatosis in the Western Hemisphere, according to Selzner et al. [17]. Homozygous Ob Zücker rats lack the brain leptin receptor and develop obesity at the age of 8 weeks due to markedly increased food intake and decreased energy expenditure. Steatosis in the Ob Zucker rats was previously determined by Serafin et al. [18], using specific lipid staining. In this study, Ob Zücker rats showed severe and macrovesicular and microvesicular fatty infiltration in hepatocytes (between 60% and 70% steatosis). The rats were housed under conditions of conventional animal facilities, with controlled temperature, humidity, and twelve hours dark/light cycles. Animals had free access to water and dry-food-standard diet, and they were distributed into different experimental groups as described below. This study was performed following the European Union Directive for animal experiments (2010/63/EU), and all the experiments were conducted in accordance with protocols approved on 14 July 2016 (No. 483116). Concurrently, they were validated by the Ethics Committees for Animal Experimentation of the University of Barcelona (Directive 483/16).

For this study, a Sham group and three experimental groups were performed, as described below. The abdomen was cut with a midline incision, and following bile duct cannulation, the portal vein and the splenic and gastroduodenal veins were ligated. After organ recovery, the livers were flushed with IGL-0 (without PEG35 and 3 mmol/L GSH), IGL-1 (1 g/L PEG35, and 3 mmol/L GSH), or with IGL-2 (5 g/L PEG35 and 9 mmol/L GSH) and stored in each solution for 24 h at 4 °C. The animals were randomly distributed as follows:

Sham: Ob Zücker rats underwent transverse laparotomy, and the silk ligatures of right suprarenal and diaphragmatic veins and hepatic artery were performed.

IGL-0: After effecting the organs collection, livers were flushed with 50 mL of an IGL-0 solution and straightaway stored in an IGL-0 preservation solution for a 24 h period at 4 °C.

IGL-1: After the organs collection, livers were flushed with 50 mL of an IGL-1 solution and immediately stored in an IGL-1 preservation solution for a 24 h period at 4 °C.

IGL-2: After the organs collection, livers were flushed with 50 mL of an IGL-2 solution and next stored in an IGL-2 preservation solution for a 24 h period at 4 °C.

After the cold storage period described, organs maintained in their corresponding preservation solutions were flushed with 20 mL of Ringer Lactate solution. Then, all samples were stored at −80 °C for subsequent biochemical analysis.

### 2.2. ATP and Succinate Determination

The measurements of ATP and succinate concentrations in liver grafts homogenized in a perchloric acid solution were performed using the ATP assay kit (Sigma-Aldrich, Merck KGaA, Darmstadt, Germany) and the succinate assay procedure (Megazyme, Wicklow, Ireland). ATP was based on a fluorometric assay, which determined ATP amount through the phosphorylation of glycerol, which resulted in the formation of a fluorometric product (excitation/emission: 535/587 nm) proportional to the amount of ATP in the sample. Results are expressed in nmol of ATP per mg of weight of fresh tissue. Succinate was utilized by Succinyl-CoA Synthetase to form an intermediate, which underwent a series of reactions. The amount of NAD^+^ formed in the last reaction pathway was stoichiometric with the amount of succinic acid. It was NADH consumption, which was measured by the decrease in the absorbance at 340 nm. Results are expressed in nmol of succinate per mg of weight of fresh tissue.

### 2.3. Oxidant and Antioxidant Assays in Liver

#### 2.3.1. Lipid and Protein Oxidation

Lipid peroxidation was measured by the Thiobarbituric Acid Reactive Substances assay, which mainly detected malondialdehyde (MDA), one of the end-products of lipid peroxidation. For this assay, livers were homogenated at 10% in RIPA (Tris-HCl 50 mM; NaCl 150 mM; NaF 5 mM; SDS at 0.1%; Triton x-100 at 1%; DOC at 1%; pH 7.4). Thiobarbituric acid (TBA) reacted with the MDA of the samples, and the formation of MDA–TBA adducts was fluorometrically measured at an excitation wavelength of 515 nm and an emission wavelength of 550 nm. The calibration curve was determined using tetraethoxypropane. Values are expressed as MDA–TBA adducts in nmol/mg protein.

The identification of oxidative damage caused to proteins was conducted via advanced oxidative protein products (AOPP) test. This protocol is founded on Witko-Sarsat assay [19], where the AOPP content is determined from a standard of chloramine-t. The formation of AOPP in the liver homogenates was spectrophotometrically measured at 340 nm. The AOPP concentration is expressed in µmol Chloramine-t/mg protein.

Carbonyl groups, as a hallmark of oxidative modification of proteins, were also measured. The carbonyl groups in the protein side chains were derivatized to 2,4-dinitrophenylhydrazone (DNP hydrazone) by reaction with 2,4-dinitrophenylhydrazine (DNPH). Derivatization with 2,4-dinitrophenylhydrazine (DNPH) was performed according to the procedure of Nakamura and Goto [20]. Briefly, a mixture was prepared by combining equal amounts (30 μg) of samples, precipitated by trichloroacetic acid, and then incubated in 10 mM DNPH in 2 N HCl at room temperature for 1 h. The DNP-derivatized protein samples were separated by polyacrylamide gel electrophoresis followed by Western blotting, as explained below.

#### 2.3.2. Antioxidant Enzymes Activity

The measurement of the antioxidant enzymes superoxide dismutase (SOD, EC 1.15.1.1), glutathione S-transferase (GSH S-T, EC 2.5.1.18), glutathione peroxidase (GSH-Px, EC 1.11.1), and glutathione reductase (GSH-R, EC 1.6.4.2) were performed with the Sigma-Aldrich Determination Kit (SOD, Cat. 19160, Sigma, St. Louis, MO, USA) and Cayman Kits (GSH S-T, 703302; GSH-Px, 703102; GSH-R, 703202, Cayman Chemical, Ann Arbor, MI, USA), following the manufacturer’s instructions. The catalytical activity of catalase (EC 1.11.1.6) was determined according to Aebi method (1984) [21], by the spectrometric analysis of the H_2_O_2_ consumption at 240 nm. Results are expressed as enzymatic activity units (U) in relation to the total protein of the samples.

The total protein content in liver (μg/mL) was determined using the Bio-Rad protein assay (Bio-Rad Laboratories, Hercules, CA, USA) based on the Bradford dye-binding method.

#### 2.3.3. GSH

Reduced glutathione (GSH) and oxidized glutathione (GSSG), and the redox ratio (GSH/GSSG) were determined following the protocol of Hissin and Hilf, modified by us [22,23]. The o-phthaldeyde (OPA) reagent was added to the samples and the standard calibration curve, so that it reacted with the GSH at pH = 8, and it was fluorometrically measured at an excitation wavelength of 350 nm and an emission wavelength of 420 nm. GSSG was detected through previous incubation with N-ethylmaleimide, which was added to GSH in order to avoid its interference. Results are expressed in nmol of GSH relative to g of fresh tissue or as the GSH/GSSG ratio.

### 2.4. Western Blotting Analysis and Quantification

The detection of DNP-protein carbonyl groups (Novus), uncoupling protein 2 (UCP2), PTEN-induced kinase 1 (PINK1), nuclear factor-erythroid 2 related factor 2 (Nrf2), heme oxygenase-1 (HO-1), NOD-, LRR-, and pyrin domain-containing protein 3 (NLRP3) (Santa Cruz Biotechnology, Heidelberg, Germany), and mitochondrial oxidative phosphorylation complexes (OXPHOS) (Abcam, Cambridge, UK) was carried through Western blotting. Liver samples were homogenated in an RIPA buffer (as described below), separated in 10% sodium dodecyl sulfate-polyacrylamide gel electrophoresis (SDS-PAGE) and plotted into PVDF membranes. After overnight blocking with an Odyssey^®^ Blocking Buffer (PBS 927-40000, LI-COR Biosciences, Lincoln, NE, USA), membranes were incubated with the primary antibody for 3 h at room temperature. Detection was performed with chemiluminescence (anti-IgG-HRP, Santa Cruz Biotechnology, Inc., Heidelberg, Germany) or fluorescence (IRDye, LI-COR Biosciences, Lincoln, NE, USA) signals exposed and quantified by LI-COR Odyssey system and the Image Studio programme (LI-COR Biosciences, Lincoln, NE, USA). Revert total protein stain, REVERT-TM (LI-COR LI-COR Biosciences, Lincoln, NE, USA), was used as a loading control [24]. (The revert development of each membrane and complete blots are shown in Figure S1 in Supplementary Materials).

### 2.5. Statistical Analysis

Data obtained are expressed as the means and SDs of each group for *n* = 4–6. The variance analysis was carried through the one-way ANOVA and Tukey’s post-hoc test for multiple comparisons in terms of significant results. A *p*-value of <0.05 was considered statistically significant. The graphs and statistic analysis were performed with GraphPad prism 7 (GraphPad software, San Diego, CA, USA).

## 3. Results

### 3.1. IGL-2 Prevents ATP Breakdown and Succinate Accumulation and Affects Mitochondrial OXPHOS Complex Expression

**Figure 1:**
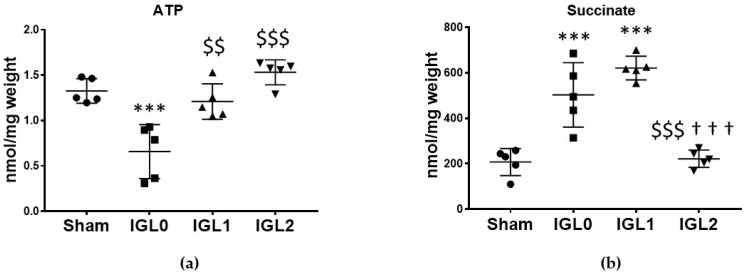
Energy metabolites as ATP (**a**) and succinate (**b**) in steatotic livers preserved (4 °C, 24 h) in the IGL-0, IGL-1, and IGL-2 solutions, vs. Sham. Bars represent mean values ± SDs of each group (*n* = 5). Differences are shown comparing groups (* vs. Sham, ^$^ vs. IGL-0, and ^†^ vs. IGL-1) according to the one-way ANOVA test and the Tukey post-hoc test (one symbol indicates *p* < 0.05; two symbols indicate *p* < 0.01; three symbols indicate *p* < 0.001).

ATP depletion and succinate accumulation is a major feature in ischemic tissues. Our results showed that after 24 h of SCS at 4 °C, ATP levels (Figure 1a) decreased significantly in the samples preserved with IGL-0 (without PEG35 and 3 mmol/L GSH) when compared to in the control group (Sham) (*p* < 0.001) and when compared to in the samples preserved in IGL-1 (1 g/L PEG35, and 3 mmol/L GSH) (*p* < 0.01) and IGL-2 (5 g/L PEG35 and 9 mmol/L GSH) (*p* < 0.001). Cold ischemia induced the accumulation of succinate, as observed in the IGL-0 and IGL-1 groups (Figure 1b). However, this accumulation of succinate was prevented in IGL-2-preserved livers.

**Figure 2:**
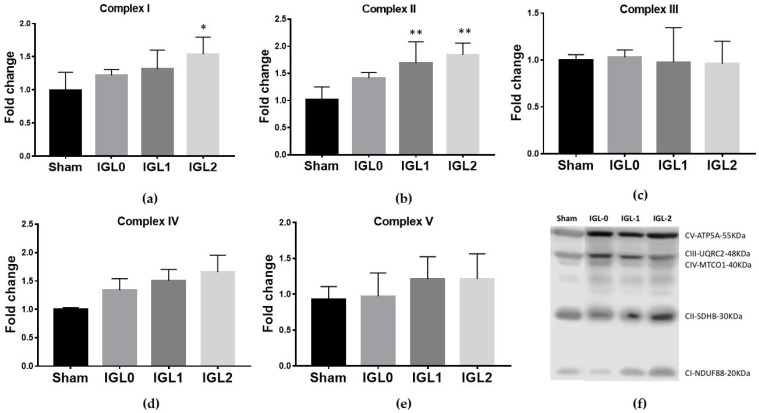
Oxidative phosphorylation complexes (OXPHOS) expression levels in complex I NADH–coenzyme Q oxidoreductase (**a**), complex II succinate–coenzyme Q oxidoreductase (**b**), complex III coenzyme Q-cytochrome C oxidoreductase (**c**), complex IV cytochrome C oxidase (**d**), complex V ATP synthase (**e**), and representative blots of OXPHOS in steatotic livers preserved (4 °C, 24 h) (**f**) in IGL-0, IGL-1 and IGL-2 solutions, vs. Sham. The bars represent the mean values ± SDs of each group (*n* = 4–6). Differences are shown comparing groups (* vs. Sham), according to the one-way ANOVA test and the Tukey post-hoc test (one symbol indicates *p* < 0.05; two symbols indicate *p* < 0.01; three symbols indicate *p* < 0.001).

Mitochondrial OXPHOS complex expression is shown in Figure 2. The oxidation of NADH and FADH_2_ and the subsequent phosphorylation of ADP to form ATP took place in the inner mitochondrial membrane through electron transport chain protein complexes (I–IV) and ATP synthase (complex V). Complex I NADH–coenzyme Q oxidoreductase expression was significantly increased in the IGL-2 group (Figure 2a). Complex II succinate–coenzyme Q oxidoreductase expression was significantly increased in the IGL-1 and IGL-2 groups (Figure 2b). Complex IV cytochrome C oxidase had an increasing tendency in the IGL-2 group without statistic difference (Figure 2d). There were no changes in the levels of hepatic mitochondrial complexes V ATP synthase (Figure 2e) and III coenzyme Q–cytochrome C oxidoreductase (Figure 2c), among groups.

### 3.2. UCP2 Expression and Mitophagy Increases in IGL-2 Preservation

**Figure 3:**
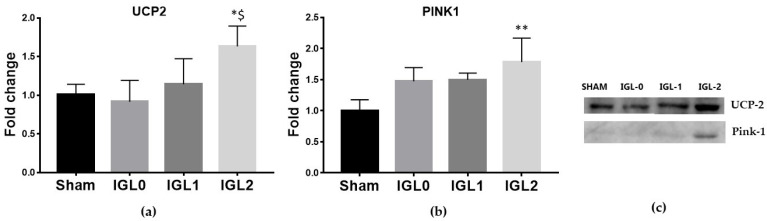
Expression levels of uncoupling protein 2 (**a**) and mitophagy marker protein PTEN-induced kinase 1 (PINK1) (**b**) in steatotic livers preserved (4 °C, 24 h) in the IGL-0, IGL-1, and IGL-2 solutions, vs. Sham. (**c**) Representative blots. The bars represent the mean values ± SDs of each group (*n* = 4–6). Differences are shown comparing groups (* vs. Sham, ^$^ vs. IGL-0) according to the one-way ANOVA test and the Tukey post-hoc test (one symbol indicates *p* < 0.05; two symbols indicate *p* < 0.01; three symbols indicate *p* < 0.001).

The mitochondrial protein UCP2 acts as an anion carrier, and it is involved in the regulation of various processes such as cellular homeostasis, oxidative stress, and cell survival. Recently, a link has emerged between UCP2 cytoplasmic accumulation and mitophagy stimulation, particularly with respect to cardiomyopathies, including IR injury [25]. In this sense, we determined whether the PEG35 and GSH addition to preservation solutions could exert a protective effect through UCP2 upregulation. The results showed a significant increase in the expression of UCP2 in those livers preserved with IGL-2 (Figure 3a). We also evaluated the expression levels of PINK1, the main regulator of mitochondrial biogenesis [26], and observed a significant increase in the PINK1 expression (Figure 3b) in those livers preserved in IGL-2.

### 3.3. Nrf2 Transcription Factor Increases in IGL-2 Solution Preservation

**Figure 4:**
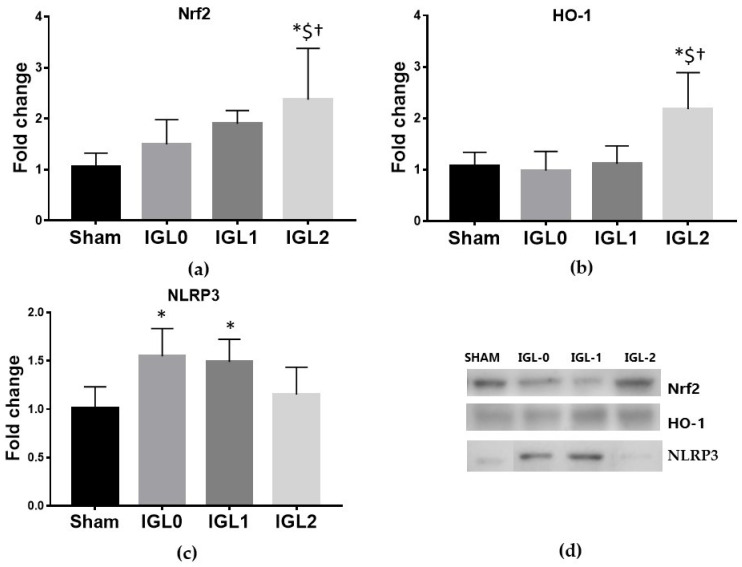
Nrf2-mediated response to oxidative stress in steatotic livers preserved (4 °C, 24 h) in the IGL-0, IGL-1, and IGL-2 solutions vs. Sham: (**a**) Nrf2 transcription factor; (**b**) HO-1; (**c**) NLRP3; and (**d**) representative blots. The bars represent the mean values ± SD of each group (*n* = 4–6). Nrf2 and HO-1 expression followed the same trend, being higher in the IGL-2 group compared to in the other groups. Differences are shown comparing groups (* vs. Sham, ^$^ vs. IGL-0, and ^†^ vs. IGL-1) according to the one-way ANOVA test and the Tukey post-hoc test (one symbol indicates *p* < 0.05; two symbols indicate *p* < 0.01; three symbols indicate *p* < 0.001).

Nrf2 acts as a transcription factor by inducing the expression of cytoprotective gene products and plays a key role in activating cellular defense mechanisms. In order to explore whether the protective effects of PEG35 and GSH are associated with the Nrf2 response, we analyzed its expression, finding a dramatically increase of the transcription factor as well as its downstream enzyme HO-1 in IGL-2-preserved livers (Figure 4a,b). Inverse relationships between Nrf2 pathways and NLRP3 inflammatory pathways have also been reported at different levels [27,28]. In this regard, the inflammasome NLRP3 expression was found to be significantly increased in IGL-0 and IGL-1 preserving livers, while no differences were found when using IGL-2 (Figure 4c).

### 3.4. IGL-2 Preservation Solution Better Protects Steatotic Livers from Oxidative Stress

**Figure 5:**
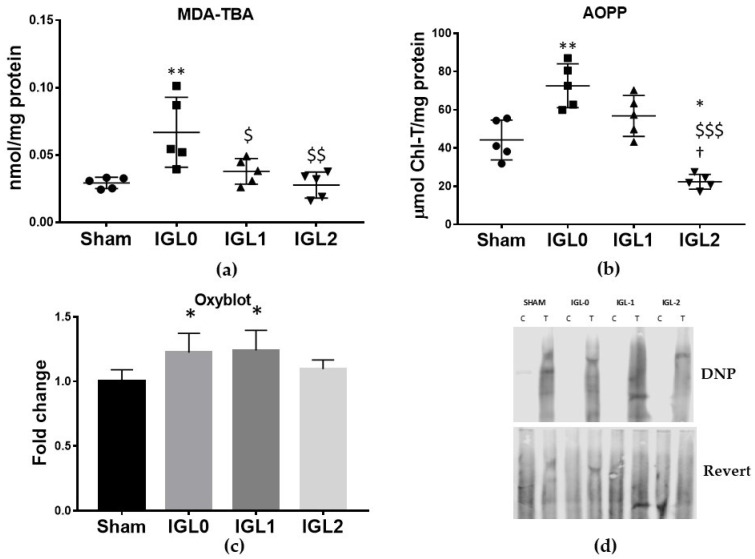
Hepatic oxidative stress damage in steatotic livers preserved (4 °C, 24 h) in the IGL-0, IGL-1, and IGL-2 solutions vs. Sham: (**a**) MDA-TBA adducts expressed in nmol/mg of total protein; (**b**) advanced oxidative protein products (AOPP) expressed in μmol chloramine-t/mg of total protein; (**c**) protein carbonyl formation (Oxyblot) analyzed by immunoblotting and expressed as a relative unit; (**d**) representative blots for protein oxidation. The bars represent the mean values ± SDs of each group (*n* = 4–6). Differences are shown comparing groups (* vs. Sham, ^$^ vs. IGL-0, and ^†^ vs. IGL-1) according to the one-way ANOVA test and the Tukey post-hoc test (one symbol indicates *p* < 0.05; two symbols indicate *p* < 0.01; three symbols indicate *p* < 0.001).

The cold preservation of steatotic livers increased oxidative stress damage, as measured by lipid peroxidation (MDA–TBA adducts) and protein oxidation (AOPP and carbonylated proteins). With the known antioxidant capacity of GSH, we investigated whether these oxidative stress markers were altered in the livers preserved with the different solutions. A significant rise on MDA–TBA adducts (Figure 5a) was observed in IGL-0 when compared to in Sham, IGL-1, and IGL-2. AOPP has been identified as a biomarker of oxidative damage to proteins, detecting dityrosine and cross-linking protein products [29]. AOPP levels (Figure 5b) significantly increased in the IGL-0 group compared to those in the Sham, IGL-1, and IGL-2 groups, while significant lower levels were observed in the IGL-2 group. Carbonylated proteins, the most general and widely used marker of severe protein oxidation, increased in the IGL-0 and IGL-1 groups when compared to in the Sham group (Figure 5c). Altogether, these results showed less oxidation of lipids and proteins in SCS livers when the increasing concentrations of PEG35 and GSH were added to the preservative.

### 3.5. Antioxidant Enzymatic Activity

**Figure 6:**
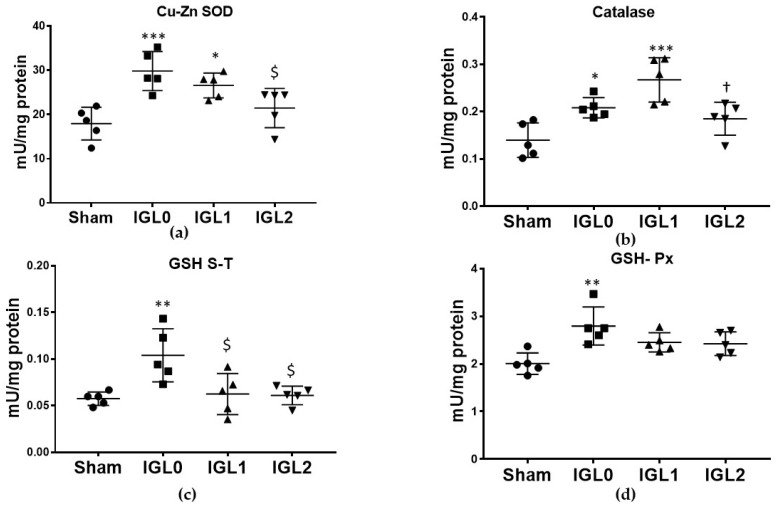
Enzymatic antioxidant capacity in steatotic livers preserved (4 °C, 24 h) in the IGL-0, IGL-1, and IGL-2 solutions vs. Sham: (**a**) superoxide dismutase (Cu–Zn SOD) expressed in mU/mg of total protein; (**b**) catalase expressed in mU/mg of total protein; (**c**) glutathione S-transferase (GSH S-T) expressed in mU/mg of total protein; (**d**) glutathione peroxidase (GSH-Px) expressed in mU/mg of total protein. The bars represent the mean values ± SDs of each group (*n* = 5). Differences are shown comparing groups (* vs. Sham, ^$^ vs. IGL-0, and ^†^ vs. IGL-1) according to the one-way ANOVA test and the Tukey post-hoc test (one symbol indicates *p* < 0.05; two symbols indicate *p* < 0.01; three symbols indicate *p* < 0.001).

Regarding antioxidant enzymatic activity, the group preserved in IGL-0 showed a higher activity in the antioxidant enzymes—Cu–Zn SOD (Figure 6a), GSH S-T (Figure 6c) and GSH-Px (Figure 6d), when compared to the Sham group. This correlated with the higher oxidative stress previously exposed in the IGL-0 group. The liver preservation in the IGL-1 solution increased the activities of Cu–Zn SOD and catalase, when compared to Sham livers, and decreased the activity of GSH S-T compared to that in the IGL-0 solution. When comparing the IGL-2 solution (with higher amounts of PEG35 and GSH) to IGL-0, the results showed significantly lower antioxidant activities for Cu–Zn SOD and GSH S-T. No significant differences were found between IGL-2 and Sham and in any antioxidant enzymatic activity, which correlated with a lower amount of oxidative stress biomarkers in livers preserved in the IGL-2 group.

**Figure 7:**
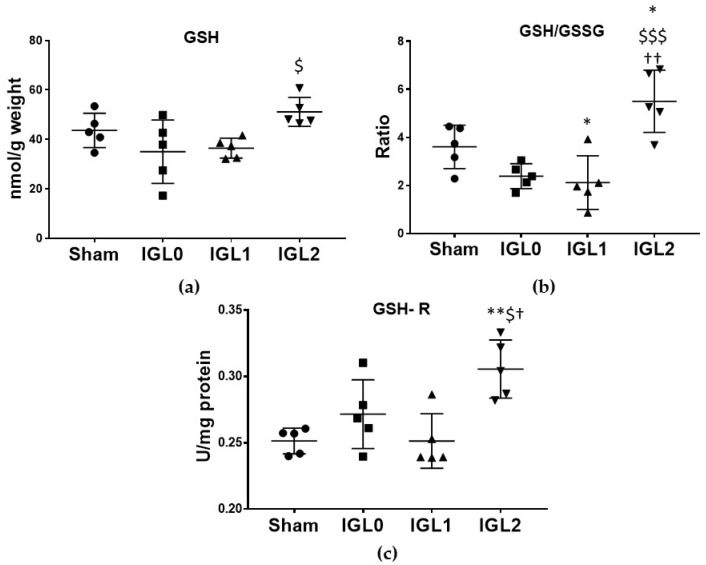
Glutathione status in steatotic livers preserved (4 °C, 24 h) in the IGL-0, IGL-1, and IGL-2 solutions vs. Sham: (**a**) reduced glutathione (GSH) expressed in nmol GSH/g fresh weight; (**b**) reduced glutathione/oxidized glutathione ratio (GSH/GSSG); (**c**) glutathione reductase (GSH-R) expressed in mU/mg of protein. The bars represent the mean values ± SDs of each group. Differences are shown comparing groups (* vs. Sham, ^$^ vs. IGL-0, and ^†^ vs. IGL-1) according to the one-way ANOVA test and the Tukey post-hoc test (one symbol indicates *p* < 0.05; two symbols indicate *p* < 0.01; three symbols indicate *p* < 0.001).

In contrast to other enzymes, the GSH-R, the enzyme responsible for reducing GSSG to GSH, showed a different pattern of activity, being significantly higher, in livers preserved in IGL-2 compared to in all the other groups (Figure 7c). This result is of great interest, since the reason why livers preserved in IGL-2 showed a significantly higher amount of GSH (Figure 7a) and a significantly higher GSH/GSSG ratio (Figure 7b) were not only attributable to higher content in the medium, but also to the increased activity of the GSH-R.

## 4. Discussion

SCS remains the standard for liver preservation, although the effects due to anaerobic metabolism during the ischemic phase result in succinate accumulation and ATP depletion. These effects have been identified as the leading cause of reperfusion injury, causing oxidative stress and tissue inflammation [16]. This is of particular relevance in the case of fatty livers, as steatosis affects the activity of oxidative phosphorylation during cold liver preservation [30].

Our work showed the protective role that PEG35 and GSH play in the prevention of energy degradation and in the modulation of the redox state in cold steatotic livers preservation when using homozygous Ob Zücker rats.

During the cold ischemia phase, ATP comes from glycolysis, a significantly less efficient route of ATP production, while some anaerobic metabolites accumulate. The selective accumulation of the citric acid succinate is a feature of the ischemia response. With the particularity that succinate can control reperfusion injury through the formation of mitochondrial ROS by reverse electron transport at mitochondrial complex I. The pharmacological manipulation of the pathways that generate succinate accumulation has been shown to improve IR injury in murine models of heart attack and stroke [31]. In this regard, it should be noted that we observed lower succinate levels in livers preserved in IGL-2. In addition, PEG35 (IGL-1 and IGL-2) maintained ATP levels in the graft. This together may suggest a protective role of these components in the preservation medium.

Mitochondrial oxidative phosphorylation activity is decreased by cold ischemia in fatty livers, contributing to the reduced capability of steatotic grafts to restore the ATP after transplantation [30]. Until now, the OXPHOS mitochondrial expression of cold-preserved fatty liver has not been addressed. We observed enhanced expression of complex I and complex II in livers preserved with PEG35 and GSH. Recently, increased expression of complexes I, II, III, and V has been reported in a murine model of cardiac IR in young mice. In contrast, the expression is lower in older individuals [32]. Therefore, the increased expression of OXPHOS components would be aimed at maintaining mitochondrial integrity under IR stress.

The protection of mitochondria is crucial to prevent IR injury [16]. In an early investigation, we found that SCS preservation with PEG35 improves mitochondrial biomarkers and the mitochondrial enzyme ALDH2 functionality [10,15]. In the present work, we found that the preservation with IGL-2 induces a significant increase in the expression of mitochondrial protein UCP2, an anion carrier involved in the regulation of various processes, such as maintaining mitochondrial membrane potential and limiting the generation of reactive oxygen species [33,34]. In fact, UCP2 silencing reduces the levels of GSH and the GSH/GSSG ratio in HepG2 cells [35]. UCP2 can also function as an oxidative stress sensor, not directly involved in antioxidant defense, but through other adaptation mechanisms [36]. Moreover, UCP2 activates IR-induced mitophagy [25], and the inhibition of mitophagy abolishes UCP2 cardioprotective effects in IR [37]. Accordingly, we found a significant increase in PINK1 expression linked to enhanced UCP2 expression in IGL-2-preserved fatty livers. This PINK1 pathway is one of the best characterized among mitophagy signaling pathways [26], and such enhanced expression is consistent with our previous results, in which we also found an increase in the mitophagy markers Beclin1 and LC3B in the fatty livers preserved with PEG35 [15].

The protective role of PEG35 is also evidenced in the Nrf2-mediated response. The activation of the Nrf2 transcription factor could prevent IR injury, both through the activation of antioxidant and anti-inflammatory pathways [38,39,40]. In our study, the preservation of steatotic livers in the IGL-2 solution increased the Nrf2 expression and that of the antioxidant system, HO-1. Nrf2 is also associated with the constitutive expression of GSH-dependent enzymes [41]. Regarding the anti-inflammatory response, the expression of the NLRP3 inflammasome is reduced. It has also been reported that Nrf2 can control mitophagy by directly binding to the promoter regions of PINK1, which would provide an advantage for cell survival [42]. Decreased NLRP3 expression, along with increased PINK1, indicates that Nrf2 could regulate the cytoprotective mechanism of IGL-2 preservation.

Nrf2 has recently been proposed as a predictive marker for organ recovery and donor expansion in human allografts [43]. Since we found that the IGL-2 solution significantly increased Nrf2 expression, we can expect the better viabilities of these steatotic grafts against IR damage after transplantation.

We have recently demonstrated how the direct effects of IGL-2 on mitochondrial ALDH2 protect the steatotic liver graft from the damaging effects of cytotoxic aldehydes generated by ROS overproduction [15]. In the present work, we found a reduction in lipid peroxidation and oxidized proteins correlated with increased concentration of PEG35 and GSH in the preservation solution. However, only the use of IGL-2 solution completely reversed the increase found with IGL-0. Carbonylated proteins decreased when fatty grafts were preserved with the combination of PEG35 and a high dose of GSH. The importance of several endogenous antioxidants has been demonstrated during ischemic heart injury in early studies [44,45]. Cardiac ischemia induces a significant increase of cytoplasmic GSH-Px activity, while superoxide dismutase activity is unmodified. The greater the oxidative damage, the greater the antioxidant enzymatic activity. In our results, the enzymatic activity was higher in the groups with higher levels of oxidation of lipids and proteins, except for the activity of GSH-R, which was the enzyme that recycled GSH from its oxidized form, increasing its activity and the GSH level in the IGL-2 group. Since ROS regulation is a therapeutic strategy for liver IR injury, decreased lipid and protein damage and antioxidant properties appear to be one of the most relevant mechanisms mediating the beneficial effects of PEG35 used in cold storage solutions.

## 5. Conclusions

The steatotic liver is especially vulnerable to IR lesions that significantly determine graft function after transplantation. An in-depth understanding of the molecular mechanisms that lead to tissue and cell damage is therefore essential. Cold fatty liver preservation with PEG35 and GSH (IGL-2 solution) maintained ATP production, decreased succinate accumulation and increased the expression of the OXPHOS complexes I and II, UCP2, PINK-1, Nrf2, and HO-1. IGL-2 protected against lipid and protein oxidation, increased the GSH/GSSG ratio and decreased the inflammasome NLRP3 expression. Taken together, its effects included protecting mitochondrial integrity, maintaining ATP levels, and reducing oxidative stress and inflammation. Further research on whether these conditions would protect the graft during the reperfusion phase would be necessary in view of its potential use in clinical transplantation.

## Data Availability

Data is contained within the article and supplementary material.

## Supplementary Materials

The following are available online at https://www.mdpi.com/article/10.3390/antiox11010158/s1, Figure S1: Membranes and loading control (Figure 2f). Figure S2: Membranes and loading control (Figure 3c). Figure S3: Membranes and loading control (Figure 4d). Figure S4: Membranes and loading control (Figure 5d).
