# Supplementary material for: PEG35 and Glutathione Improve Mitochondrial Function and Reduce Oxidative Stress in Cold Fatty Liver Graft Preservation
Source: Antioxidants (Basel). 2022 Jan 14;11(1):158. doi: 10.3390/antiox11010158 (PMC8772919; doi:10.3390/antiox11010158)
Supplement: Supplementary file 1 [file antioxidants-11-00158-s001.zip › antioxidants-1524792-supplementary.pdf]

OXPHOS S

IGL 0 1 2

Loading control for OXPHOS

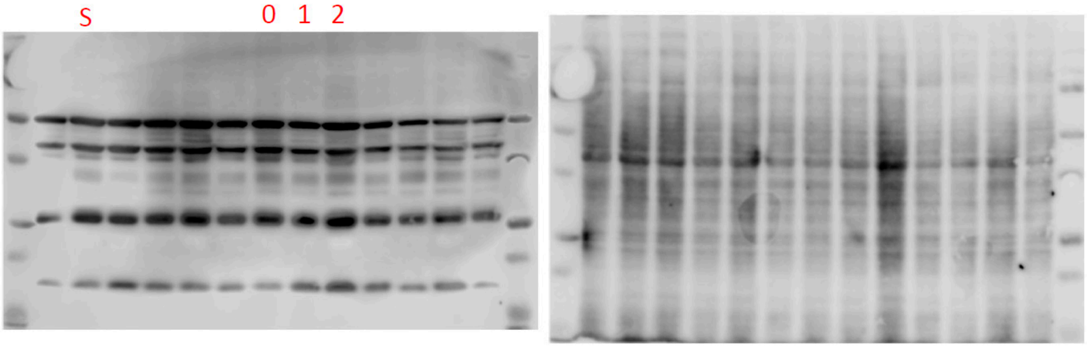

Lane 3: Sham
Lane 8: IGL-0

Lane 9: IGL-1
Lane 10: IGL-2

Figure S1. Membranes and loading control (Figure 2f).

UCP2

IGL IGL

S 0 1 2

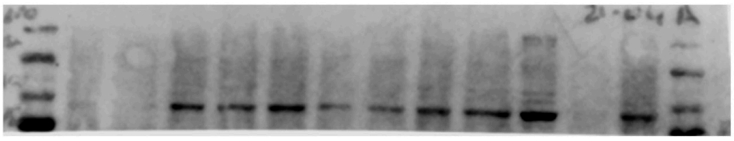

Lane 4: Sham
Lane 5: IGL-0

Lane 10: IGL-1
Lane 11: IGL-2

Pink1

IGL IGL

S 0 1 , 2

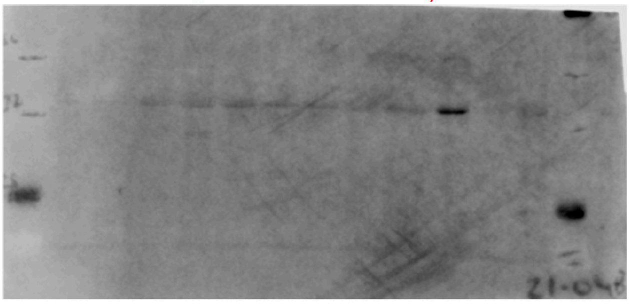

Lane 4: Sham
Lane 5: IGL-0

Lane 10: IGL-1
Lane 11: IGL-2

Loading control for UCP2 and Pink1

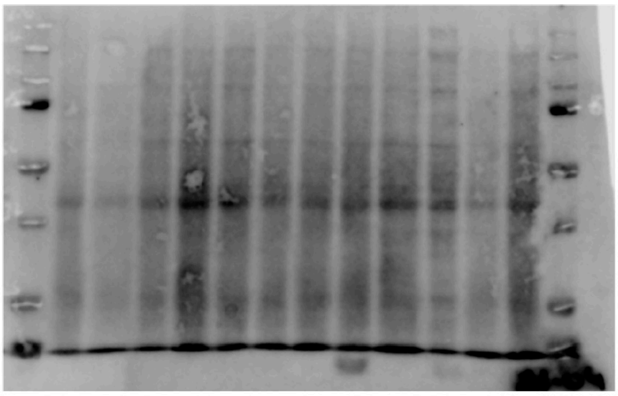

Lane 4: Sham
Lane 5: IGL-0

Lane 10: IGL-1
Lane 11: IGL-2

Figure S2. Membranes and loading control (Figure 3c).

Nrf2 IGL
S 0 1 2

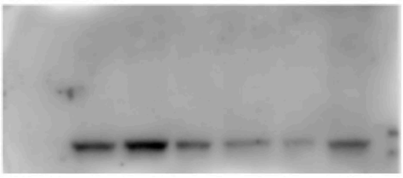

Loading control for Nrf2

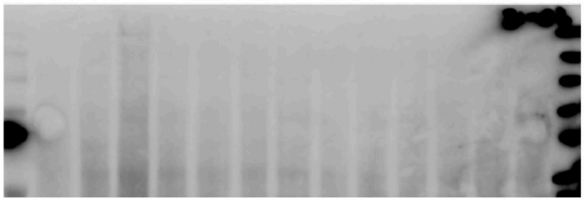

Lane 4: Sham
Lane 5: IGL-0
Lane 6: IGL-1
Lane 7: IGL-2

HO-1 IGL
S 0 1 2

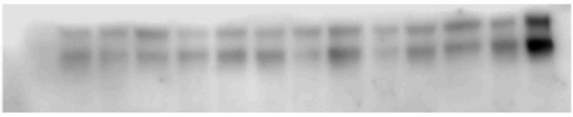

Loading control for HO-1

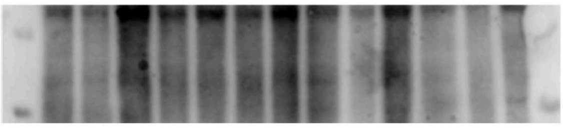

Lane 4: Sham
Lane 5: IGL-0
Lane 6: IGL-1
Lane 7: IGL-2

NLRP3 IGL IGL
S 0 1 2

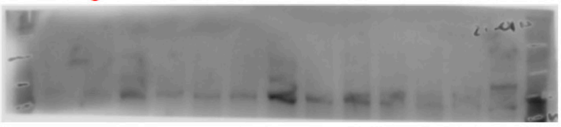

Loading control for NLRP3

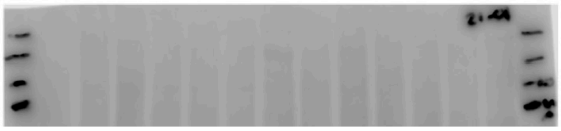

Lane 3: Sham
Lane 6: IGL-0
Lane 7: IGL-1
Lane 12: IGL-2

Figure S3. Membranes and loading control (Figure 4d).

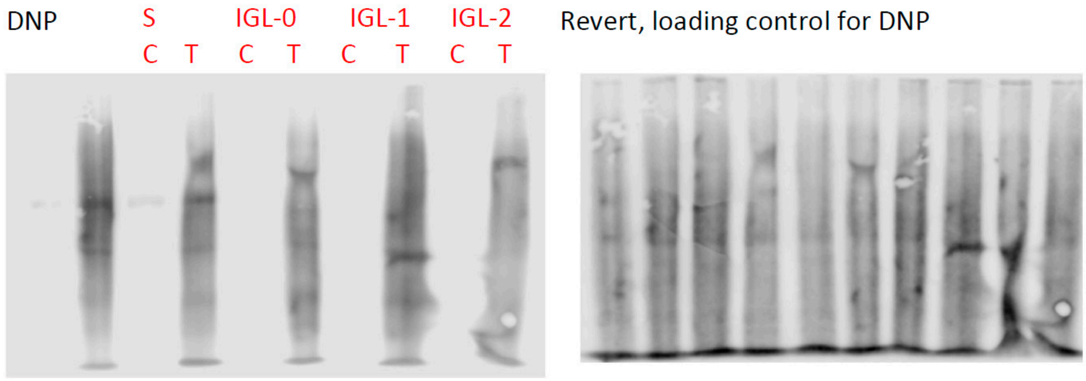

Lanes 3,4: Sham

Lanes 5,6: IGL-0

Lanes 7, 8: IGL-1

Lanes 9,10: IGL-2

Figure S4. Membranes and loading control (Figure 5d).
